# Spatio-Temporal Optimization of Perishable Goods’ Shelf Life by a Pro-Active WSN-Based Architecture

**DOI:** 10.3390/s18072126

**Published:** 2018-07-02

**Authors:** Daniela De Venuto, Giovanni Mezzina

**Affiliations:** Department of Electrical and Information Engineering, Politecnico di Bari, 70125 Bari, Italy; giovanni.mezzina@poliba.it

**Keywords:** WSN, shelf life, automated platform, ZigBee, Bluetooth

## Abstract

The waste in the perishable goods supply-chain has prompted many global organizations (e.g., FAO and WHO), to develop the Hazard Analysis and Critical Control Points (HACCP) protocol that ensures a high degree of food quality, minimizing the losses in all the stages of the farm-to-fork chain. It has been proven that good warehouse management practices improve the average life of perishable goods. The advances in wireless sensors network (WSN) technology offers the possibility of a “smart” storage organization. In this paper, a low cost reprogrammable WSN-based architecture for functional warehouse management is proposed. The management is based on the continuous monitoring of environmental parameters (i.e., temperature, light exposure and relative humidity), and on their combination to extract a spatial real-time prediction of the product shelf life. For each product, the quality decay is computed by using a 1st order kinetic Arrhenius model to the whole storage site area. It strives to identify, in a way compatible with the other products’ shelf lives, the position within the warehouse that maximizes the food expiration date. The shelf life computing and the “first-expired first-out” logistic problem are entrusted to a Raspberry Pi-based central unit, which manages a set of automated pallet transporters for the displacement of products, according to the computed shelf lives. The management unit supports several commercial light/temperature/humidity sensor solutions, implementing ZigBee, Bluetooth and HTTP-request interfaces. A proof of concept of the presented pro-active WSN-based architecture is also shown. Comparing the proposed monitoring system for the storage of e.g., agricultural products, with a typical one, the experimental results show an improvement of the expected expiration date of about 1.2 ± 0.5 days, for each pallet, when placed in a non-refrigerated environment. In order to stress the versatility of the WSN solution, a section is dedicated to the implemented system user interfaces that highlight detecting critical situations and allow timely automatic or human interventions, minimizing the latter.

## 1. Introduction

About 1.3 billion tons of food (i.e., 1/3 of the overall food for human use) and $35 billion per year (in the US) [1], are some of the numbers that characterize the socio-economic losses caused by the global problem of Food Losses and Waste (FLW) [1]. FLW concerns the whole food-chain, but it is important to distinguish between the “food losses” that refer to the pre-consumer losses and the “food waste” that is a post-consumer reality. While in this latter case, the improvement depends largely on consumers’ sensitization and common sense, in the former one the food losses minimization is entrusted to a protocol, known as Hazard Analysis and Critical Control Points (HACCP) [2], legislated by an agreement between the Food and Agriculture Organization (FAO) and the World Health Organization (WHO). This protocol follows the perishable goods supply-chain and identifies critical points in the “farm-to-fork” stages, providing useful parameters for the optimal monitoring of the product quality, minimizing the pre-consumer losses. The HACCP identifies the temperature as the most relevant factor for maximizing the average life of products, imposing some boundaries in order to realize product-dependent cold chains [2]. If perishable products are stored or transported in environments which are above the temperature limits, rapid microbiological growth takes place leading to spoiled goods and consequent economic damage [3].

In this context, shelf life (SL) has become a pillar concept to describe the current state of product quality, as stated in its definition provided by the Institute of Food Science and Technology: “the period of time under defined conditions of storage, after manufacture or packing for which a food product will remain safe and be fit for use” [4].

There are several predictive SL models [5,6], which identify the amount of product degradation during the ageing process, basing their computing on temperature, humidity and illumination [5].

By combining the environmental real-time monitoring capability of the Wireless Sensor Networks (WSN) with the integration, on portable devices and platforms, of predictive SL models, it becomes possible to detect interruptions in the cold chain, and act in advance to compensate abnormal SL failure [6,7].

In this paper, a WSN-based architecture for the real-time environmental monitoring and the fully automatic management of pallets in a smart storage site for agricultural products (in our case study, pallets of tomatoes) is detailed. The proposed architecture addresses, together, a number of still unsolved challenges [8] such as:

- Continuous monitoring of the goods, with the possibility to follow the pallets during the transit.
- Implementation of a specific pallet’s SL prediction model extended all over the storage site. It guarantees the identification of the storage location, inside the warehouse, that optimizes the specific SL. Integration on the central control unit of a Quality Controlled Logistic (QCL) algorithm. It implements a first-to-expire first-out (FEFO) warehouse management [9].
- Pallets’ automatic displacement according to the SL prediction model and QCL algorithm outcomes.
- Easy re-programmability of the platform by in loco operations or by remote actions.
- Low cost, easy installation and operation.

The monitoring and the management of the automated warehouse are based on the continuous acquisition of the key environmental parameters: temperature (T), light exposure (L) and relative humidity (H). Then, a monitoring algorithm has been developed based on the product quality degradation via a 1st order kinetic Arrhenius model. The algorithm identifies, compatibly with the other products’ SL, the position that maximizes the food expiration, preparing the system for the FEFO logistic problem.

The SL computing, as well as the sensor interfacing, are realized on a Raspberry Pi 2 B+ (RPi) that represents the central control unit. Through a set of Python scripts, the unit analyzes the shelf lives and manages a set of automated robots for product displacement, maximizing the SL of each single product.

The RPi-based system has been designed to support several commercial light/temperature/humidity (L/T/H) sensors solutions, implementing Zigbee, Bluetooth and HTTP-request communication protocols.

The paper is structured as follows: Section 2 details and compares the state of the art in food safety monitoring and certification using WSNs. Section 3 outlines the here implemented WSN-based architecture focusing on the predictive SL model and the QCL management algorithm. Section 4 describes the automated pallet displacement operations. Section 5 presents experimental results and Section 6 concludes the paper.

## 2. State of the Art

The advances in the WSN technology concerning the number of manageable sensing nodes, as well as the power consumption optimization for sensor systems (continuous monitoring), jointly with the cost reduction of coordination platforms (e.g., Raspberry, Arduino, ARM controller, etc.) have led to several literature works in the context of reliable monitoring of perishable goods [10,11,12,13,14,15]. Most of the existing solutions, which comprise the abovementioned system features, focus on the application of new signal acquisition methods for the efficient recovery of sparse signal samples (e.g., compressed sensing) [10], or propose new web-based monitoring systems that remotely communicate with WSNs [11]. In work [10] the sensor node integrates a microcontroller, a temperature sensor and a GPRS remote transmission module. The temperature sensor shows an accuracy of ±0.5 °C (in an operative range going from −55 °C to 125 °C). No product useful life prediction is here done. The above- mentioned web-based solution [11] is used to measure plant and environment parameters in a greenhouse (e.g., leaf temperature, temperature or humidity).

To collect the data, the system exploits a tree-topology ZigBee-based WSN with 20 sensor nodes, five router nodes, and one coordinator (sink node) placed in the greenhouse. The system integrates a GPRS module connected with the coordinator via a RS-232 serial port to transmit the received data to the monitoring center. A web-based management subsystem to acquire and analyze the data via the Internet completes the architecture. In [12], an IoT approach to monitor post-harvest losses is proposed, using low cost platforms, namely Raspberry Pi as the server and Arduino + DHT11s (temperature sensors with accuracy of ±2 °C) as sensing nodes. The approach exploits machine learning to predict losses for each product in a warehouse and request human intervention to send the pallet out of the stocking site before the others (e.g., FEFO). The authors in [13] propose RFID smart tags for traceability and cold chain monitoring of foods. The smart active tag on each pallet (item), integrates light, temperature and humidity sensors, with a microcontroller, memory chip, antenna for RFID communications with the reader/writer and the required power supply system (usually batteries). Despite the number of proposed systems [10,11,12,13,14,15] for perishable goods monitoring, only few solutions are designed in the aim of maximizing the product shelf life, reducing the losses [14,15]. These solutions operate statically on the stocking site, turning on/off refrigerators (or ventilation systems), exposing the goods to proper light or acting on humidity [14] by dehumidification or a reverse process, depending to the product to be preserved. Nevertheless, solutions that merge reliable monitoring systems, prediction models and low cost platforms for a proactive goods’ shelf life maximization, in a non-refrigerated warehouse, are not yet described in the literature. Following the latest trend of logistics, e.g., automated robots for pallet movement [14,15], and embedding shelf life prediction models in a WSN-based monitoring architecture, the proposed work aims to address this challenge.

## 3. The WSN-Based Architecture

**Figure 1:**
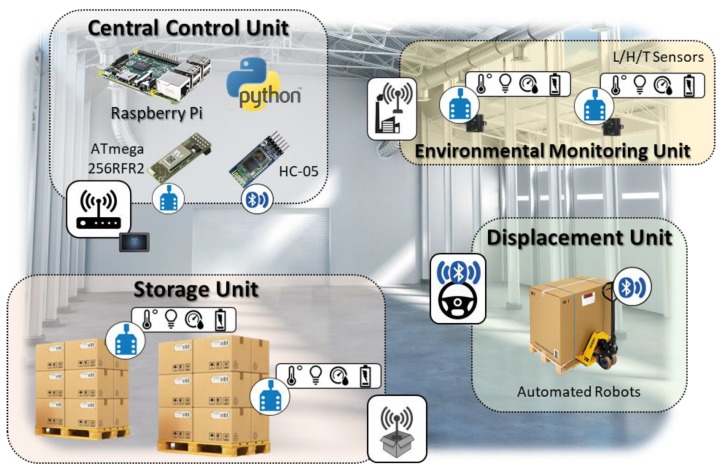
Top-level representation of the overall implemented architecture.

Figure 1 shows the top-level representation of the here proposed architecture. It schematizes a WSN-based architecture for functional warehouse management, through the continuous monitoring of environmental parameters. The automated smart system analyzes these parameters and combines them to reach a spatio-temporal optimization of the products’ SL. The system realizes this condition, acting directly on the pallets’ positions. The WSN-based architecture is composed of four main functional units: (i) Central Control Unit, (ii) Environmental Monitoring Unit, (iii) Storage Unit and (iv) Displacement Unit.

The Central Control unit consists of a Raspberry Pi 2 B+ working with Raspbian Jessie. It is equipped with an ATmega256RFR2 (Microchip Technology Inc. Chandler, AZ, USA), for the ZigBee communication and an HC-05 for the Bluetooth interfacing. The RPi implements a set of Python 2.7 scripts for the SL computing, FEFO management and automated robot dispositions. This unit is the core of the system and it is able to interact with each single secondary unit.

The Environmental Monitoring Unit consists of L/H/T sensors that acquire statically the data from a specific position of the storage room, sending them to the Central Unit via ZigBee. The Central Unit uses these data to realize a heat map of the storage site.

The Storage Unit is composed of the pallet to be monitored and a dedicated L/H/T node placed on the surface. It communicates via ZigBee with the Central Unit. The dedicated node is used to precisely define the product shelf life, because the use of parameters derived mathematically from the Environmental Monitoring Unit, can lead to an avoidable propagation of errors [16].

The Central Control Unit collects data from the Storage and Environmental Monitoring units and implements a predictive SL computing on the chosen pallet. The calculation, repeated on each available position, is useful to identify the optimal site for a specific pallet. Once this place is identified, the control unit opens a Bluetooth communication channel with a dedicated pallet transporter (Displacement Unit—Figure 1).

The transporter is driven step-by-step in its navigation by the RPi-based control unit along a pre-set virtual lattice. The transporter embeds a dedicated hardware programmed for the automatic movements, curves handling and motors power management.

Each node also provides a battery level indication and a working state flag. If the battery level is under the 5%, or the working flag is set to ‘0’, the central unit stops acquiring from the specific node and derives mathematically the data in that uncovered point. Simultaneously a warning is sent to the system manager (human component) asking for intervention.

As a proof of concept, the WSN-based monitoring and management system has been implemented in a real-life context by using ten ZigBee nodes, equipped with temperature, relative humidity and light exposition sensors. Two of these were applied on the pallet to be monitored (Storing Unit—Figure 1), while the remaining eight were bonded on the storing room walls (Environmental Monitoring Unit—Figure 1). The nodes that compose the Environmental Monitoring Unit are placed in such a way as to create a 5 × 5 array: 2 for each available wall edge (right, left, up and down considering a room cross-section).

The implemented Displacement Unit consists of an acrylic car prototype, equipped with Arduino UNO and HC-05 (Bluetooth). A dedicated and easy reprogrammable (via USB) script was uploaded on the ATmega328 microcontroller, containing the servomotors direction for the steering (PWM management) and the DC motor activation, depending of the tasks provided by the Central Control Unit. Even if in the areas in which the automated robots operate, no obstacles are typically present, the automatons are equipped with ultrasonic sensors (e.g., HC-SR04), to avoid crashes with human components or pallets out of their correct place.

### 3.1. Sensing Platform

The chosen L/H/T sensor consists of an NXP JN5164 microcontroller (NXP Semiconductors Netherlands B.V., Eindhoven, The Netherlands) interfacing a 10-bit resolution ADC, for sensor data and digitization, and a Digi XBee 802.15.4 RF module for ZigBee protocol data transmission.

The ZigBee protocol is preferable respect e.g., Bluetooth, when the primary importance specifications concern: (i) the low power consumption (Battery Life-ZigBee: >1 year, Bluetooth: 1 week), (ii) the number of nodes to be monitored (ZigBee: 65,000, Bluetooth: 8), (iii) the flexibility in the network topology [7,17,18,19]. Despite these advantages, the data rate of the ZigBee is low, 250 kb/s (best case @ ISM 2.4 GHz), but it is enough for our application.

Each sensor node uses three 1.5 AA batteries and, complete of a plastic shell, has the size: 6.85 cm × 6.35 cm × 3.30 cm for a total weight of 160 g. The transmitting power stays under 1 mW, allowing the node to be powered for 1.5 years if the read-sleep cycle is: 1 reading/30 s, and 2.5 years if read-sleep cycle is: 1 reading/60 s.

Finally, an adaptive calibration process has been done on the temperature readings, identifying a measures-based correction factor variable around −4 °C, matching the sensor producer reading guidelines [20]. Table 1 summarizes the performances of the node sensors and the dedicated modules described above.

**Figure 2:**
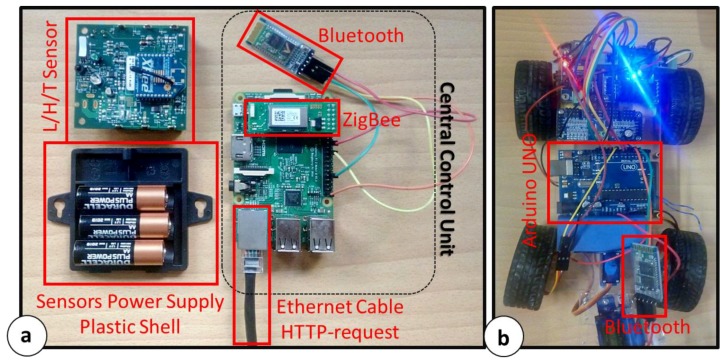
Architecture components: (**a**) L/H/T sensor and Raspberry Pi 2 Model B+ equipped with ZigBee and Bluetooth modules; (**b**) Prototype car driven by Arduino UNO core and Bluetooth.

Figure 2 shows the described architecture components. In particular, Figure 2a shows a single the L/H/T sensor equipped with ZigBee Modules and the plastic shell footprint. It also shows the Central Control Unit (Raspberry Pi 2 Model B+) equipped with ZigBee and Bluetooth devices. It is notable that the RPi is connected to a router via Ethernet cable (HTTP-Request). The prototype car used for the proof of concept is showed in Figure 2b.

### 3.2. Communication Management

**Figure 3:**
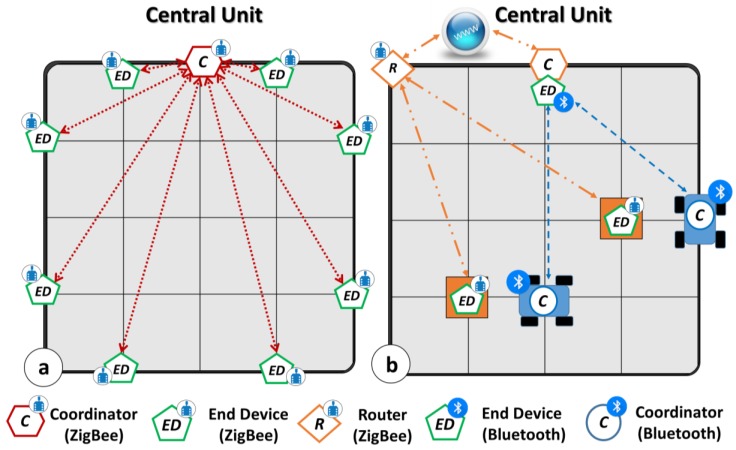
Communication Protocols: (**a**) Multipoint ZigBee Network for Environmental Control Unit; (**b**) Router-based ZigBee Network for pallets monitoring and Bluetooth point-to-point Displacement Unit management.

Three main communication networks have realized the proof of concept for the proposed architecture as sketched in Figure 3. They manage the Environmental Control, the Storage and the Displacement Unit, respectively.

The Environmental Control Unit is managed through a multipoint ZigBee-based network (Figure 3a—Red lines) in which the Central unit acts as Coordinator, while the L/H/T sensors act as End Devices. As shown in Figure 3a, the Central Unit interrogates the sensor data with the prescribed timing and waits for the response. No interactions between sensors are allowed by the Coordinator as reported in Figure 3a. Here, the red lines represent the connection between the End Devices and the Central Unit, which manages the Environmental Control Unit nodes. In order to cover all the communication possibilities, in Figure 3b the Storage Unit communicates with the Central Unit (brown solid lines) through a commercial standalone routing gateway (e.g., Digi ConnectPort X4 H Nema). The gateway locally operates as Coordinator, but globally it can be considered as a Router. The gateway connects two L/H/T sensors placed on the pallets that work as End Devices. The router acquires the data from the sensors and sends them to a personal cloud website. The Central Unit acts as global Coordinator capturing the data via HTTP-request, using reserved credentials on the dedicated Digi site (Figure 3b—Orange lines).

Finally, a point-to-point Bluetooth-based network handles the Displacement Unit (Figure 3b—Blue lines). In this case, the Central Unit operates as End Device communicating, when asked by the network Coordinator, a string that contains: (i) the address of the transporter selected for the displacement and (ii) the destination position. In this net, all the robots act as Coordinator, asking to the Central Unit the transmitted string content respecting a time multiplexing.

## 4. Shelf-Life Prediction

### 4.1. Shelf Life Prediction 1st Order Kinetic Model

The Central Control Unit has the role of processing the environmental parameters, aiming to identify critical conditions for the SL management.

In particular, the control unit implements a reprogrammable algorithm for the SL assessment focusing on fresh-cut vegetables parameters (e.g., tomatoes).

In this context, it has been proven [3,20] that the food quality is affected by several physical, chemical and microbiological reactions that can be related, through mathematical models, to the food color and firmness, that are themselves linked to temperature, humidity and light exposition, via empirical function/constants known as kinetic parameters.

A number of literature studies [3,4,5,6,7,8,9,10,11,12,13,14,15,16,17] have showed that, among the extracted environmental variables, the temperature is the parameter that significantly emphasizes worsening food quality.

The empirical law [21,22] that describes the temperature dependence in a simple chemical reaction is the Arrhenius law. It has been proven to be very worthwhile in chemical kinetics and relates the rate constant *k* of a reaction with the absolute temperature T, through the following equation:

(1) k(T)=We−EaRT

with W: pre-exponential factor, Ea: activation energy and R: gas constant. The Ea can be evaluated as the energy barrier that the molecules need cross, in order to be able to react, while W represents the rate constant at which all the molecules have sufficient energy for the reaction.

Since the Arrhenius law parameters are typically determined with non-linear regression of empirical data, such as the one in [6,21,22], in the following, we will insert the kinetic parameters, provided by a tomatoes-centered study, in a general formula for k rate derivation, suitable for any temperature:

(2) $$k\left(T\right)=k_{\mathrm{ref}}e^{-\frac{\mathrm{Ea}}{R}\left(\frac{1}{T}-\frac{1}{T_{\mathrm{ref}}}\right)}$$

with k_ref_: k value at the dedicated reference temperature T_ref_.

According to [22] for climacteric fruit, the initial firmness is considered as a good indicator of the products ripeness and it is recommended in the shelf-life assessment of fresh-cut tomatoes as stated by several works in the field [21,22,23,24].

For this reason, this proof of concept bases its functioning on the tomatoes’ firmness as the only parameter for the quality level determination [22]. However, the here implemented kinetic model is suitable for the parallel evaluation of other kinds of indexes, such as color, dehydration (loss of weight) and microbial contamination.

In the firmness evaluation context, the extracted data, analyzed in the following, were inspired to the standardized measurements in [22], where the force necessary to cause a deformation of 3 mm (with a speed of 0.02 mm/s) was recorded using a Zwick Universal Testing Machine. The extraction of the decay rate k is operated on empirical basis as stated in [24].

A first order approximation about the product quality is given by Equation (3):

(3) $$c=c_{\mathrm{eq}}+\left(c_{0}\right)-e^{-k\left(T\right)t}$$

where c is the measured quality factor, c_0_ is the initial quality value, c_eq_ is the quality factor at a specific equilibrium value, t is the storage time and k is the rate determined by Equation (2).

Then, known a specific quality factor c′ at a given time t, the SL can be calculated as:

(4) $$\mathrm{SL}\left(t\right)=\frac{1}{k}\ln\left(\frac{c^{\prime }}{c_{\mathrm{eq}}}\right)$$

Together, Equations (2)–(4), allow calculating the products’ remaining life, estimating with a first order model its degradation in the presence of temperature changes. A compendium of the parameters implemented in the proposed monitoring system, extracted tomatoes-centred studies [6,21,22], are shown in Table 2.

**Figure 4:**
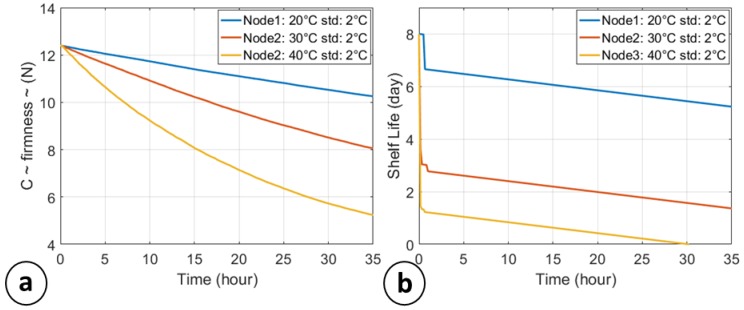
Food quality decay: (**a**) changes in the quality factor c (firmness [N]) of tomato stored at different temperatures; (**b**) Shelf life modulation of tomatoes at different temperature.

Figure 4 shows the change in firmness (N) of tomatoes stored at three different (and controlled) temperatures: 20 °C ± 2 °C (blue line), 30 °C ± 2 °C (red line) and 40 °C ± 2 °C (yellow line). The data that compose the plot have been acquired each 60 s, but are reduced to 1 read/500 s for plotting purposes.

The initial shelf life, imposed by the kinetic parameters in Table 2, is about 8 days, but all the nodes show lower SL: 6.9 days for the Node120 °C, about 3 days for the Node2 @ 30 °C SL and a remaining life of about 1.5 days for the third node at 40 °C.

The model used to describe a food quality parameter, such as the firmness, is generic and remains valid for every kind of product and quality parameter (e.g., color, weight loss, enzymatic activity, etc.). The only difference concerns the empirically derived kinetic parameters, which characterize the specific product.

It is notable that the implemented prediction model exhibits a certain degree of approximation, showing a linear degradation of the SL. Since the temperature sensors shows an accuracy of ±2 °C, the degradation rate k can assume, according to the Equations (1) and (2), values between k^−^(T_1_) and k^+^(T_2_) with T_1_ = T + 2 °C and T_2_ = T − 2 °C. For the same reason, the SL ranges between SL^−^(k(T_1_)) and SL^+^(k(T_2_)) according to Equations (3) and (4).

### 4.2. Implemented Monitoring and Control Protocol

**Figure 5:**
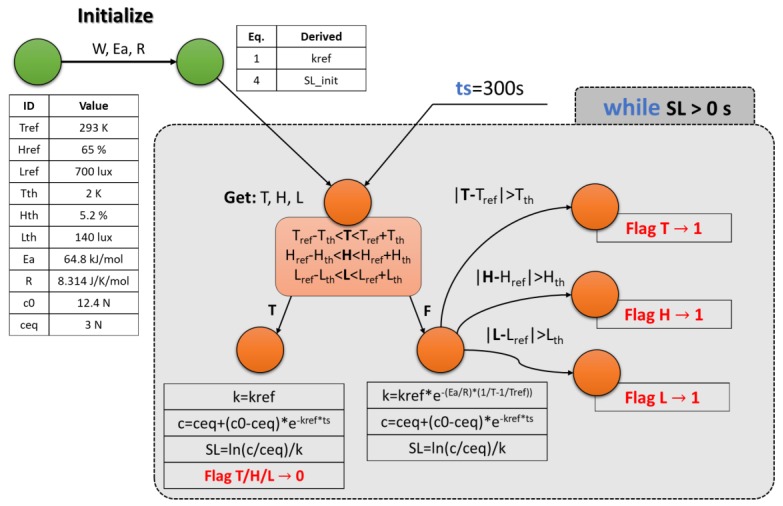
Monitoring and control protocol flowchart from the initialization to the control flag definition. The initialization stage is shown by green circles, while the cycle body is represented by orange circles.

As described in Section 4.1, the implemented SL algorithm is focused on the temperature effects, but a series of control flags are set according to the guidelines of the psychrometric [6,19] (effect of the relative humidity on a specific perishable good) and light exposure effects on the product quality decay [19]. Figure 5 summarizes the main steps of the implemented monitoring and control protocol, on a single product (e.g., pallet, or stack of pallets [3]). They consist of:

1. Upload, on the system interface, the T/H/L reference values and sensors accuracies. The initialization phase will derive the k_ref_ [21] (according to Equation (1)) and the initial shelf life (Equation (4)).
2. Start the computation in *the while cycle*, driven by the condition: SL > 0. The while repeats the computation with a fixed sampling time (ts). In this example ts = 300 s (e.g., 1 read/5 min).
3. Get T/H/L values from sensors interfaces and compare them with the reference ones (T/H/L_ref_), taking into the account the sensors accuracies (T/H/L_th_).
4. If the recommended conditions are respected, the system uses k_ref_ as k(T) in Equations (3) and (4) for calculating quality parameter c and computing SL, respectively. If a threshold, or more than one, is surpassed (potentially incorrect storage conditions), the rate k is calculated according Equation (2), the quality parameter c and the SL are derived, respectively, via Equations (3) and (4). Then, the values that overcame the respective thresholds, activate a dedicated flag for an external intervention request.

The warnings provided by the system appear in the frontal panel of the RPi-based central control unit. Automatic corrective actions for the conditions recovery in terms of humidity and light exposition are not yet integrated in the present system and are left to the human component.

Differently, in the temperature control context, a fully automatic environment management has been implemented, ensured the SL maximization of all the pallets inside the smart warehouse.

### 4.3. Quality-Controlled Logistic Algorithm

The algorithm described in Section 4.1 and Section 4.2, in the presented form, allows the monitoring and the control of each pallet (or pallet stack) independently by the surrounding conditions. It is suitable in static situations, in which a pallet cannot be moved from its initial position [6,19], but it is not able to provide a prediction of the position, inside a storage room, that maximizes its lifetime. For this aim, the proposed Central Control Unit embeds a quality controlled logistic (QCL) algorithm that exploits the data provided by the L/H/T sensors from the Environmental Control Unit, to realize a heatmap of the whole storage room. Then, in principle, considering a single pallet to be preserved, it is assumed to virtually place the same in all the possible sites. Then, since the temperature is mathematically derived for each available site, for the same a certain number of SL values are derived (number of available storing sites minus 1). The resulting-linked SL matrix provides a quantifiable effect of the potential displacement on the product remaining life. Maximizing all the SL for all the pallets within a warehouse it is possible to implement a first-to-expire first-out (FEFO) management system. The QCL algorithm working principle can be briefly summarized in five main steps:

1. The algorithm virtually derives, on the storage room surface, an M_1_ × M_2_ lattice (the dimensions are arbitrary, in this case M_1_ = M_2_ = M = 5 as shown by the background matrix in Figure 3). The N_p_ pallets to be managed (with N_p_ < M^2^ − 3) are placed on N_p_ row-column different intersections.
2. The system identifies each pallet with a number (ID) and derives an *encumbrance matrix* (EM) used to define the navigation path of the selected pallet to be moved. It allows pre-calculating the path in order to avoid collisions with other pallets.
3. The system extract two M × M matrices, named **T_ref_** and **T_amb_**. The former, **T_ref_** ∈ℝ**^M,M^**, consists in a matrix that contains the temperature reference value [6,19,20]. For sake of clarity, in the following T_refi,j_ will identify the element in the i-th row and j-th column. The second matrix, **T_amb_** ∈ℝ**^M,M^**, contains the temperature values acquired by sensors and the mathematically derived ones. In the proposed example, eight values of T are directly extracted by the sensors, according to the end devices in Figure 3a: {T_amb1,2_, T_amb1,4_, T_amb2,1_, T_amb2,5_, T_amb4,1_, T_amb4,5_, T_amb5,2_, T_amb5,4_}. The other matrix elements are mathematically derived by a contiguous elements average, as proposed in literature [25].
4. The temperature matrices lead to the definition of N_p_ overlapped 2D quality matrices, which constitute a 3D matrix **C** ∈ℝ**^M,M,Np^**, with C_i,j,p_ the quality that a specific p-th would have, if it were in i-th row and j-th column position.

The algorithm controls sequentially all the pallets and analyzes the effective C_i,j,p_ value (row and column indexes of real position of the pallet) of the considered pallet. If C_i,j,p_ of the monitored product shows a firmness decrease between two consecutive measurements, of C_th_ = 2.68 × 10^−5^ N/s (10 min @ 24 °C), the system activates the displacement operations. The quality factor threshold C_th_ is reprogrammable and two thresholds are preset by default: 10 min @ 26 °C → 3.184 × 10^−5^ N/s and 10 min @ 24 °C → 2.68 × 10^−5^ N/s.

5. Since the algorithm has the **C** and **T_amb_** matrices, it can derive the shelf life 3D matrix **SL** ∈ℝ**^M,M,Np^** with SL_i,j,p_ the shelf life of a specific p-th pallet would have, if it were in i-th row and j-th column position. The displacement operations ask for identifying (with the right order) the pallets movements that maximize the overall shelf life in the warehouse.

**Figure 6:**
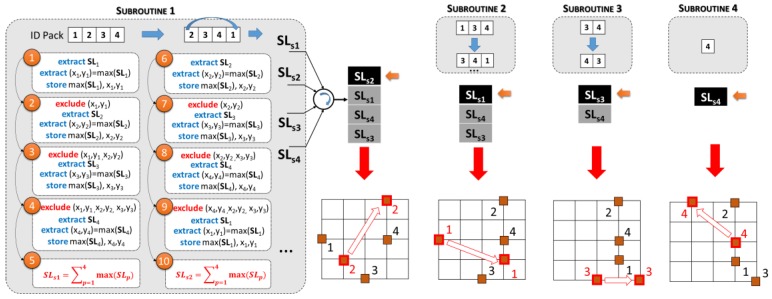
QCL Algorithm with expanded subroutines and displacement actions example.

For the sake of clarity, the routines on which the algorithm is based are schematically reported in Figure 6, considering the displacement of four pallets. During the 1st subroutine (Subroutine 1—Figure 6):

(a) Firstly, the pallet with ID = 1 is selected and the coordinates (x_1_, y_1_) that maximize the SL of the first pallet are identified on the **SL_p=1_** map.
(b) The coordinates (x_1_,y_1_) are excluded from the computation, and the point (a) is repeated on the **SL_p=2_** matrix. Then, the algorithm has identified the maximum SL for the pallet with ID = 2 and the coordinates that ensure it.
(c) The steps (a) and (b) are repeated for the four pallets. Then, a sum of all the four **SL_p_** is done defining the SLs_1_ parameter, that represent the overall shelf life of the warehouse content if the first evaluated pallet is the ID = 1, and the last is the 4th.
(d) All the points from (a)–(c) are repeated, shifting the indexes vector of a single position on the left. In this case the first ID is 2, and the last ID is 1. The routine defines the SLs_2_. Then the iterations are repeated considering as first ID the 3, and as last the 2, and so on (Figure 6).
(e) After 20 iterations the system has four values of SLs: {SLs_1_, SLs_2_, SLs_3_, SLs_4_}. Then, it sorts these values in a decreasing order. The highest overall SL (e.g., SL_s2_ in the example) identifies the “best pallet” to be displaced.

After the displacement, the moved node is excluded by the computing, and a similar subroutine (Subroutine 2) repeats the working principle from the point (a) to the (e), but operating on three IDs. After 12 iterations the second “best pallet” is identified. Overall after 39 recursive iterations, all the pallets are disposed in regions that ensure the maximum overall shelf life.

### 4.4. Displacement Unit Management

When the Central Control Unit defines all the coordinates according to the QCL algorithm, it also identifies a step-by-step path for a specific pallet in order to avoid collision with other pallets. The system drives in each displacement the pallet by using a specific string mounted on a Bluetooth transceiver in receiver mode (End device—Section 3.2 Figure 3b). The string is composed of:

S=uint8([89 add_btT add_btP 89],[90 init_coord 90],[91 final_coord 91])

with 89, 90, 91 random separators, add_btT is the address of the selected transporter, while add_btP is the address of the selected; init_coord are the initial coordinates and final_coord are the final ones. The separators uniquely define the parts of the messages, avoiding errors in the communication.

When the QCL algorithm ends the computation and the Central Control Unit has defined the paths for the pallets displacement, the Raspberry Pi commutates its system role from Coordinator in a ZigBee Network to End Device in a Bluetooth one. Then it sends in “broadcast” a message of READY, and then waits for the coupling.

The first transporter operates a pairing with the RPi-connected HC-05 and reads the string. If add_btT field contains a different address, the transporter closes the communication with the control unit, otherwise reads the remaining string. In the first case, the second transporter acts as Coordinator, asking for add_btT field.

**Figure 7:**
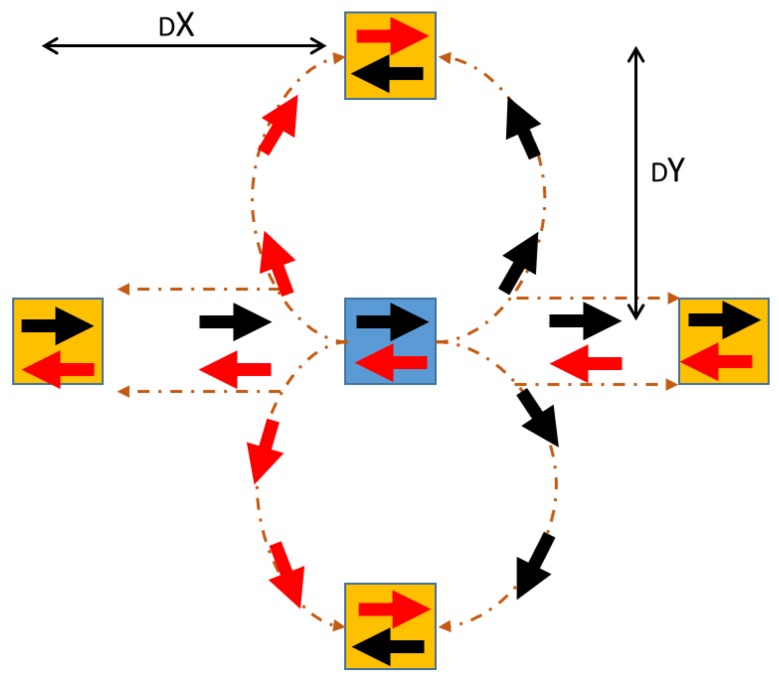
Unified automatic transporter movement circuit. Black and red arrows show all the available direction starting from a specific initial position (blue square).

When the needed transporter is found, an on-board Arduino UNO script analyzes each part of the string identifying the pallet initial position and the “final coordinates”. Since the transporter movement are fully driven step-by-step on the virtual lattice, the final coordinates provided by the control unit are simply the next coordinates to be reached and thus differ by one point on the x-axis or y-axis. It allows one to fully define all the available movements as summarized by the following pseudocode and Figure 7:

1. **BT paired**        #Coherent add_btT, add_btP
2. Define Max1, Max2   #Maximum row and column indexes
3. Define Cp, Rs     #Cp: central angular position for wheels, Rs: Rotation angle (right and left) to be subtracted or added to Cp.
4. Init x1 = x2, y1 = y2    #Initial Position (1), Final Position (2)
5. Init dX, dY = 0      #Difference between x-axis and y-axis points during displacement
6. Init Z = 0         #FSM state
7. Init SM0,SM1       # Driving Variables for servomotor (Steering)
8. Init DCm0,DCm1     # Driving Variables for DC Motors
9. Read String
   → verify Separator: if separator = 90
       → x1, y1            #Previous Final position
        if x1 > Max1 & y1 > Max2  #if Out of range, wait for next communication
          break
       → x2, y2            #Next Position
        if x2 > Max1 & y2 > Max2  # if Out of range, wait for next communication
          break
10. Check all separators
11. dX = x2 − x1; dY = y2 − y1; S = (dX + dY)
12. define dX1,dX0, dY1,dY0**dX (or dY)****{dX1,dX0} or {dY1,dY0}**000101−111
13. if S > 1         # wrong step-by-step path (variation on X and Y axis)
     break
14. # Driving Bit Computing
15. SM1 = (dX0 **OR** dY0) **AND** (**NOT** dX1) **AND** (**NOT** dX0) **AND** (Z **XOR** dY1)
16. SM0 = (dX0 **OR** dY0) **AND** (**NOT** dX1) **AND** (**NOT** dX0)
17. DCm1 = (dX0 **OR** dY0) **AND** (**NOT** dY1) **AND** (**NOT** dY0) **AND** (**NOT** (dX1 **XOR** Z))
18. DCm0 = (dX0 **OR** dY0) **AND** ((**NOT** dY1) **AND** (**NOT** dY0)) **OR** ((**NOT** dX1) **AND** (**NOT** dX0))
19. Z = ((dX0 **OR** dY0) **AND** (((**NOT** Z) **AND** (**NOT** dX1) **AND** (**NOT** dX0)) **OR** (Z **AND** (**NOT** dY1) **AND** (**NOT** dY0))) **OR** (**NOT**(dX0 **OR** dy0) **AND** Z)
20. # Steering Angle Management
21. write to Servomotor angle → Cp-Rs*SM0*(2*SM1-1) #Angular position of servomotor
22. # Steering Angle Management**IN1****IN2****Event**0 (1)0 (1)Keep the position01Go Ahead10Go Backward
23. write to DC motors → IN1 ← DCm1, IN2 ← **NOT**(DCm1) **AND** (DCm0) #Motor Management

Figure 7 shows the unified movement circuit that takes into account all the available movements and initial direction of a selected transporter (via add_btT). A blue square represent the *instantaneous* starting point, while the orange ones represent the available target positions. It is clear that all the suitable positions differ of a single step along the x or y axis. Figure 7 shows that the implemented algorithm is suitable for all the final direction of the transporter (e.g., red or black arrow). The final direction determines the servomotor and DC motors driving bits (SM0, SM1, DCm0, DCm1).

## 5. Results

The system described above has been fully implemented and analytically validated, realizing a proof of concept in an empty University Lab room with size [6.3 m × 6.7 m], virtually divided into a 5 × 5 lattice. The eight L/H/T sensors described in Section 3.1 were placed on the walls with a uniform height from the floor of *h* = 1.2 m.

Considering a top view of the room such as the one in Figure 3, the x-axis has been placed on the wall with length l_1_ = 6.3 m, while the y-axis on the wall with length l_2_ = 6.7 m. Annotating the sensors coordinates in the form S_n_(x_n_, y_n_), with n the ID of the specific sensor, the monitoring nodes are placed in: S1(1.26 m, 0 m), S2(3.78 m, 0 m), S3(0 m, 1.34 m), S4(0 m, 4.02 m), S5 (1.26 m, 6.7 m), S6(3.78 m, 6.7 m), S7(6.3 m, 1.34 m), S8(6.3 m, 4.02 m). The internal room heat-map has been derived following the procedure in Section 4.3. Finally, for the proof of concept, only a single transporter was used (Figure 2b). All the results in the following section have been extracted from an environmental monitoring of 10 days and 11 h. In the following will be provided experimental results about the kinetic model approximations, Central Control Unit users’ interface and monitoring panel, and quantitative shelf life optimization.

### 5.1. Kinetic Model Approximations Assessment

Since the internal room heat-map is mathematically derived by an average procedure described in Section 4.3, before proceeding to the sensor placement a quantitative evaluation of the absolute temperature error has been done. This evaluation allows identifying the maximum intersensor distance (Isd) that ensures a temperature error below the sensors’ accuracies even if the temperature on a specific position is derived from a contiguous element average.

**Figure 8:**
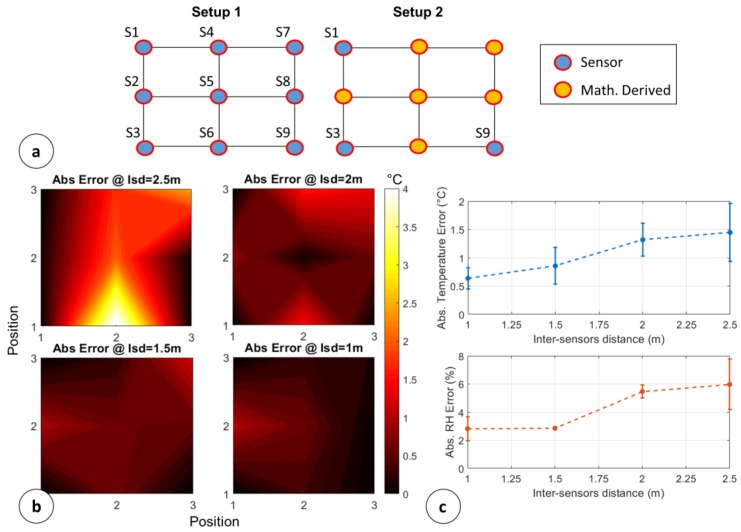
Absolute errors considering mathematically derived values: (**a**) Ground truth setup (Setup1) vs. mathematically derived setup; (**b**) Absolute temperature errors with variable Isd; (**c**) Average and standard deviations of temperature and RH errors vs Isd.

For this aim, a section of the room was divided in a 3 × 3 lattice and two setups were realized, as shown by Figure 8a. Setup 1 covers all the available positions with physical sensors realizing a kind of ground truth of the temperature, while Setup 2 covers only three positions with sensors and the remaining six ones are mathematically determined.

The assessment of the absolute temperature error (ε) was done according to Equation (5):

(5) $$\varepsilon =\left|T_{s1,i,j}-T_{s2,i,j}\right|@\mathrm{fixed}\;\mathrm{Isd}$$

where $T_{s1,i,j}$ is the temperature in the (i,j) lattice position for the Setup 1 and the $T_{s1,i,j}$ is the temperature in the (i,j) lattice position for the Setup 2. Sixteen monitoring events for each evaluated Isd have been realized in order to extract a statistical behavior. The Isd was ranged from 1 m to 2.5 m with step 0.25 m. For sake of clarity, Figure 8b shows the absolute error of a single run for Isd = 1 m, 1.5 m, 2 m, 2.5 m, highlighting the expected decreasing trend when the sensors become close among each other. Figure 8c shows the average and standard deviations of temperature and RH errors versus Isd. The statistical behavior shows that the sensors cannot be placed further than 2.5 m in order to ensure a temperature error < 2 °C and a RH error < 8%. If the sensors’ accuracy changes, another characterization needs to be done. Moreover, in general, it is possible to extract an empirical rule about the number of needed sensors to cover the storing room with a temperature error lower than the sensors accuracy:

(6) $$N_{s}=\mathrm{round}\left\{\frac{l_{1}+l_{2}}{\mathrm{Isd}@\mathrm{std}\left(\varepsilon \right)>\mathrm{Acc}}\right\}$$

with Isd@std(ε)>Acc, the inter-sensors distance which ensures an error lower than the sensor accuracy, derivable by a characterization like the one in Figure 8c.

### 5.2. Central Control Unit Interface

**Figure 9:**
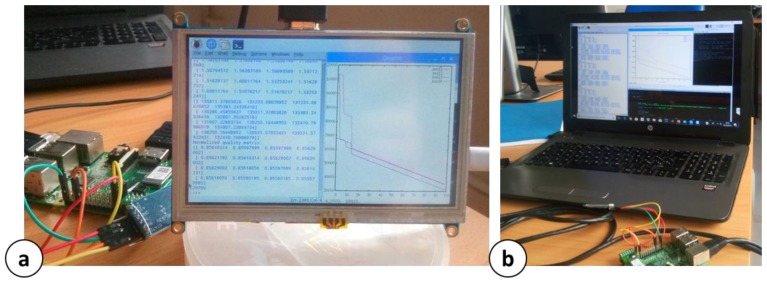
Snapshots of Central Control Unit possible setups: (**a**) Local Interface through 7″ LCD; (**b**) Dedicated interface on a personal computer through Ethernet connection.

Figure 9 shows two snapshots of the possible (and tested) Central Control Unit interface configurations during the SL monitoring. Figure 9a shows a local interface in which a 7″ LCD touch screen is connected via HDMI to the RPi, reporting in real time the SL numerical assessment and a SL chart for specific selected positions, considering a single pallet. Figure 9b shows a more complex panel hosted on the screen of a personal computer. The RPi is connected to the PC via Ethernet. In this way, the HTTP request action can be done by using a network bridge directly on the computer, which hosts the control system.

**Figure 10:**
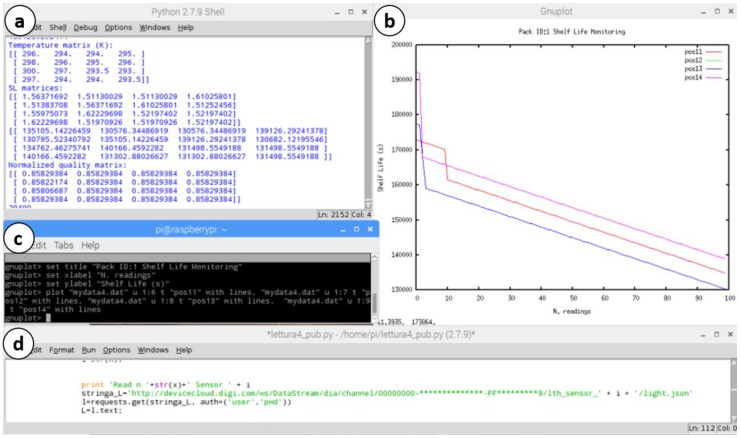
Screenshot of RPi-based Central Control Unit display: (**a**) Numeric monitoring of SL, C, T, etc. (**b**) Chart of SL monitoring of the selected positions for a specific pallet; (**c**) Chart settings panel; (**d**) HTTP-request panel for cloud reading.

Figure 10 expands the Central Control Unit panel, highlighting four main parts:

- Numerical evolution panel, which reports in real-time the SL, quality (C), temperature and information about the displacement (Figure 10a)
- Chart of SL in some selected positions (e.g., (1,1), (1,2) … (1,4)), for a specific pallet (e.g., Pack ID: 1), as shown in Figure 10b.
- A gnuplot panel (Figure 10c) that allows managing the data to be plotted (SL, C, Temperature, different ID pack).
- HTTP request Login interface (Figure 10d) for the sensors data extraction, if the router (gateway) sends them in cloud. The HTTP request is enabled by compiling a set of Python coding rows [26]. For instance, to extract the temperature data the rows are:
  >>string_T='http://devicecloud.digi.com/ws/DataStream/dia/channel/*ID Connect Port*/lth_sensor_' + i + '/temperature.json'
  >>T=requests.get(string_T, **auth=('user','pwd')**)

### 5.3. Pro-Active WSN Performance

**Figure 11:**
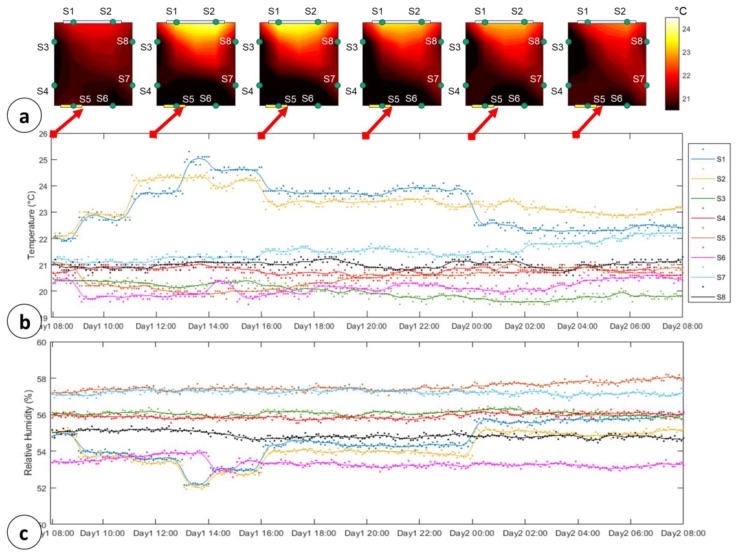
Sensors readings: (**a**) Testing room heat-map evolution during 24 h with step: 2 h; (**b**) 24 h Temperature readings by n = 8 sensors (dots) and OLS estimations (solid line); (**c**) 24 h Humidity readings by n = 8 sensors (dots) and OLS estimations (solid line).

For the validation of the proposed WSN-based system, the testing room has been monitored for about 10 days and 11 h. The placement of the environmental sensors inside the room is sketched in Figure 11a. Here, it is reported a heat-map of the room during 24 h monitoring (Day1: 8.00–Day2: 8:00). With green dots are reported the L/H/T sensors, a blue rectangle defines a window (l_3_ = 4.3 m), and the yellow one represents the door of the room.

*Control loop*. Typical profiles of temperature and RH readings during a span of 24 h are shown in Figure 11b,c. The figures show the real measurements (dots) and an ordinary least squares (OLS) approximations of the reading (solid line). The waveform trends displayed in Figure 11b,c represent well the typical behavior of the temperature and RH inside the room during all the testing duration. Indeed, the temperature measurements on 10 days returned, on average, a value of 21.3 °C ± 1.2 °C, with minimum temperature revealed on S3 and S6. The RH recorded in the room in the same time span was 55.73% ± 3%, with minimum humidity on S1, S2 and S6. The adaptive calibration of the fudge factor on the temperature sensors ensured a measure dispersion around the OLS-derived profile lower than 1.5 °C.

Figure 11 shows a typical temperature increment of 2.8 °C ± 0.8 °C from 9.00 to 16.00 on S1 and S2. These overheating cycles are due to the sunlight irradiation through the window (the sensors were not directly exposed to the sunlight). These increases fall to the average room temperature with a delay of about 0.1 °C/h. Finally, as expected the RH follow inverse trend w.r.t. the temperature one, showing decreses of about 2.5% when 3 °C temperature increments occur.

*QCL Algorithm and SL monitoring*. For the QCL algorithm validation, four pallets have been initially placed at the coordinates reported in Table 3. A typical monitoring system [10,11,12,13,14,15,19] would leave the pallets in their own initial position, sending warnings or asking for human intervention. In any case, the overall shelflife reached by all the pallets inside the testing room would be 20.871 days, with the expected expirations for pallets 1 and 4 below 5 days (with a predicted loss of −3 days/24 h considering the profile in Figure 11).

The proposed algorithm intervenes on the pallets positions by SL priority-based movements as described in Section 4.3. Table 3 shows the number of displacement operated for each pallet, and the number of priority flags (first pallet to be displaced in order to optimize the overall SL—Section 4.3).

**Figure 12:**
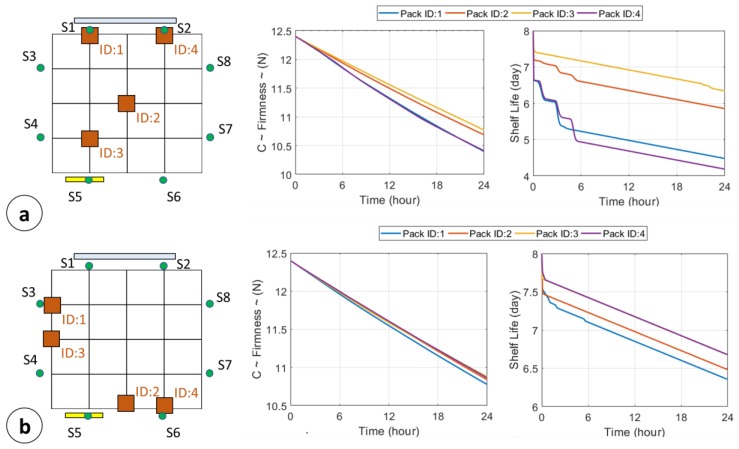
Shelflife Optimization: (**a**) Initial position of the four pallets, firmness decay and SL prediction along the 24 h; (**b**) Final position of the four pallets, firmness decay and SL prediction along the 24 h.

The last column of the Table 3 summarizes the path used by the transporter to move the pallet from the initial coordinates to the final one. Initial pallets positions and final ones are shown in Figure 12a,b, respectively. Figure 12a,b also show, on the right, the quality factor (firmness) decays and the SL predictions along the 24 h, for all the monitored nodes. From the figures is possible to extract an improvement of the overall SL of about 5.3 days.

## 6. Conclusions

In this paper, an automated infrastructure for perishable goods supply chain monitoring and their functional management has been presented. It exploits wireless sensor network technologies for goods monitoring and a parallel “real-time” processing for the shelf life prediction and maximization. The low cost of the overall platform and its easy implementation offer a suitable solution for continuous monitoring of the goods, from the farm to the fork, opening to the possibility of packaging monitoring also during the transit. Indeed, e.g., RPi Central Control Unit can manage only a certain number of pallets, and it can be made mobile and transportable, in order to follow the pallets along the whole supply chain.

Moreover, the proposed system offers an effective tool for the reduction of the waste and losses in the supply chain by the integration of Quality Controlled Logistic algorithm that ensures the maximization of the product shelf life and manages the first-to-expire first-out stack. The recorded improvements on agriculture products management show an increment of about 1.2 days (15% of maximum product useful life) of the expected expiration date. In the context of pallets management, the proposed proof of concept showed the logic network for the complete management of autonomous transporters, here based on low-cost platforms.

The re-programmability of the Central Control Unit allows one to implement more complex shelf-life prediction methods, or simply to modify the kinetics parameters in Table 2. Indeed, the approach can be used for the bacterial concentration assessment, due to the dependence from the monitored parameters. Finally, the proof of concept proposed here is therefore just a demonstration of smart warehouse system feasibility, but future perspectives will concern the performance assessment of the proposed model in real storage conditions, as well as the improvement of the automated pallets management architecture.

## Figures and Tables

**Table 1:** L/H/T Sensors and dedicated modules performance.

Sensor/Module	Specification	Value	
**Temperature**	Range	−18 °C to +55 °C	
	Sensor Accuracy	±2 °C	
**Relative Humidity**	Range	0 to 95% RH	
	Sensor Accuracy	±3.5% RH	
	Interchangeability	±5% (≤59% RH), ±8% (>59% RH)	
**Ambient Light**	Bandwidth Range ^1^	360 nm to 970 nm	
	Luminance Range	1–1000 lux (±20%)	
**Transmission Module (ZigBee)**	RF Data Rate	250 kbps	
	Sight Range	40–120 m	
	Operative frequency	ISM 2.4 GHz	
**Power Supply Module**	Reading cycle	1 reading/30 s	1 reading/60 s
	Battery Life	1.5 years	2.5 years

^1^ Wavelength of peak sensivity: 570 nm.

**Table 2:** Kinetic Parameters for firmness tomatoes SL modulation [6,19,20].

Variable	Description	Value
**k_ref_**	Rate constant of firmness decay @ 20 °C (s^−1^)	2 × 10^−1^
**Ea**	Activation Energy (kJ/mol)	64.8
**c_0_**	Initial Quality factor (N)	12.4
**c_eq_**	Quality factor at the equilibrium (N)	3

**Table 3:** Pallets Management by Implemented QCL Algorithm.

Pack ID	Initial Coord.	Num. Displacements	Priority	Path
1	(1.26 m, 0 m)	4	2	**(1.26 m, 0 m)** → (1.26 m, 1.34 m) → (1.26 m, 2.68 m) → (0 m, 2.68 m) → **(0 m, 1.34 m)**
2	(2.52 m, 2.68 m)	2	0	**(2.52 m, 2.68 m)** → (2.52 m, 5.36 m) → **(2.52 m, 6.7 m)**
3	(1.26 m, 5.36 m)	2	0	**(1.26 m, 5.36 m)** → (0 m, 5.36 m) → **(0 m, 2.68 m)**
4	(3.78 m, 0 m)	4	2	**(3.78 m, 0 m)** → (3.78 m, 1.34 m) → (3.78 m, 2.68 m) → (3.78 m, 5.36 m) → **(3.78 m, 6.7 m)**
